# A Variable Sampling Interval Synthetic Xbar Chart for the Process Mean

**DOI:** 10.1371/journal.pone.0126331

**Published:** 2015-05-07

**Authors:** Lei Yong Lee, Michael Boon Chong Khoo, Sin Yin Teh, Ming Ha Lee

**Affiliations:** 1 School of Mathematical Sciences, Universiti Sains Malaysia, 11800 Penang, Malaysia; 2 School of Management, Universiti Sains Malaysia, 11800 Penang, Malaysia; 3 School of Engineering, Computing and Science, Swinburne University of Technology (Sarawak Campus), Sarawak, Malaysia; Tianjin University, CHINA

## Abstract

The usual practice of using a control chart to monitor a process is to take samples from the process with fixed sampling interval (FSI). In this paper, a synthetic $\bar{X}$ control chart with the variable sampling interval (VSI) feature is proposed for monitoring changes in the process mean. The VSI synthetic $\bar{X}$ chart integrates the VSI $\bar{X}$ chart and the VSI conforming run length (*CRL*) chart. The proposed VSI synthetic $\bar{X}$ chart is evaluated using the average time to signal (*ATS*) criterion. The optimal charting parameters of the proposed chart are obtained by minimizing the out-of-control *ATS* for a desired shift. Comparisons between the VSI synthetic $\bar{X}$ chart and the existing $\bar{X}$, synthetic $\bar{X}$, VSI $\bar{X}$ and EWMA $\bar{X}$ charts, in terms of *ATS*, are made. The *ATS* results show that the VSI synthetic $\bar{X}$ chart outperforms the other $\bar{X}$ type charts for detecting moderate and large shifts. An illustrative example is also presented to explain the application of the VSI synthetic $\bar{X}$ chart.

## Introduction

A control chart is probably the most technically sophisticated tool among the basic Statistical Process Control (SPC) problem-solving tools to achieve process stability by reducing variability in the process. Variability exists in all processes and it is the tendency of a change occurring in a process. As a consequence of variability, no two products coming from the same process are the same. Recently, many researchers have contributed to the area of control charts, such as [1,2,3,4,5,6,7], to name a few. The traditional Shewhart $\bar{X}$ chart is commonly used to detect large mean shifts in manufacturing and service processes. However, this chart only gives a quick detection of large shifts but responds slowly to small and moderate shifts. Hence, numerous researches were made to improve the performance of the Shewhart $\bar{X}$ chart by enhancing the chart's sensitivity to detect small and moderate mean shifts.

Combining charts is not a new procedure in the literature of control charts, see for example, [8,9,10,11,12,13]. Wu and Spedding [13] introduced the combined Shewhart $\bar{X}$ and conforming run length (*CRL*) charts, which is called the synthetic $\bar{X}$ control chart. Numerous studies on synthetic control charts have been made by researchers in recent years. Wu et al. [14] presented the combined synthetic $\bar{X}$ and $\bar{X}$ chart, where this chart produces an out-of-control signal when a sample mean falls beyond the limits of the $\bar{X}$ chart or when the synthetic $\bar{X}$ chart signals. The synthetic double sampling $\bar{X}$ chart proposed by Khoo et al. [15] substantially reduces the out-of-control average run length (*ARL*_1_) and average number of observations to signal (*ANOS*) by nearly half, as compared with the synthetic $\bar{X}$ and double sampling $\bar{X}$ charts. Zhang et al. [16] studied the run-length performance of the synthetic $\bar{X}$ chart with unknown process parameters as the actual parameters are rarely known in practice. Khoo et al. [17] provided an optimal design of the synthetic $\bar{X}$ chart using the median run length (*MRL*) criterion while Yeong et al. [18] studied the economic design and the economic statistical design of the synthetic $\bar{X}$ chart using loss functions. The economic statistical design is different from the economic design as the former includes statistical constraints in its design.

An adaptive control chart involves varying at least one of the chart's parameters, such as the sampling interval, sample size or the width constant of control limits. Varying the sampling interval between samples is an alternative method adopted for a quicker detection of an out-of-control process as compared with the conventional fixed sampling interval (FSI) Shewhart $\bar{X}$ chart [19]. Costa [20] proposed taking variable sample sizes (VSS) from a process at FSI so that the chart outperforms the Shewhart $\bar{X}$ chart for detecting moderate process mean shifts. The variable sample size and sampling interval (VSSI) procedure incorporating ideas of the variable sampling interval (VSI) and variable sample size (VSS) approaches presented by Costa [21], is substantially more effective for detecting moderate process mean shifts compared with the VSI $\bar{X}$ and VSS $\bar{X}$ charts. Costa [22] extended the study of the Shewhart $\bar{X}$ chart by incorporating variable parameters (VP), where the sample size, sampling interval and factor controlling the width of the action limits, are all varied. The results showed that the variable parameters $\bar{X}$ chart is more powerful than the Cumulative Sum (CUSUM) $\bar{X}$ chart for detecting shifts in the process mean. Furthermore, numerous findings of the VSI control charts showed that these charts are substantially more efficient than the traditional FSI control charts. For instance, see [23,24,25,26,27].

The concept of varying at least one of the control chart’s parameters has been extended to adaptive type synthetic control charts. Huang and Chen [28] and Chen and Huang [29] developed adaptive synthetic *S* and synthetic *R* charts, respectively, by incorporating the VSI feature, for a quick detection of the process standard deviation. Another adaptive synthetic control chart with the VSI feature is the synthetic Max chart proposed by Chen and Huang [30], for jointly monitoring the process mean and standard deviation. To the best of the authors’ knowledge the adaptive synthetic charts that exist in the literature are mainly those mentioned above. The VSI synthetic $\bar{X}$ chart for the mean is still not in existent in the literature. Therefore, in this paper, the VSI synthetic $\bar{X}$ control charting procedure is proposed. All synthetic charts consist of two sub-charts which are the basic sub-chart at hand and the CRL sub-chart. As the VSI synthetic $\bar{X}$ chart is a type of synthetic chart, it also comprises two sub-charts, namely the VSI $\bar{X}$ sub-chart (basic sub-chart at hand) and the CRL sub-chart. Steps for computing the optimal charting parameters of the proposed chart are explained. It is shown that the VSI synthetic $\bar{X}$ chart surpasses the other $\bar{X}$ type charts, in terms of average time to signal (*ATS*). An illustrative example is provided to explain the construction of the proposed chart.

The organization of this paper hereafter is as follows: Section 2 discusses several $\bar{X}$ type charts considered in the performance comparison. The details and properties of the VSI synthetic $\bar{X}$ chart are presented in Section 3. Section 4 suggests an optimal design of the VSI synthetic $\bar{X}$ chart to minimize the out-of-control *ATS*. Performance comparisons between the proposed chart with the $\bar{X}$, synthetic $\bar{X}$, VSI $\bar{X}$ and Exponentially Weighted Moving Average (EWMA) $\bar{X}$ charts are shown in Section 5. Section 6 provides an illustrative example to show the application of the VSI synthetic $\bar{X}$ chart. Finally, conclusions are drawn in Section 7.

## An Overview of Several $\bar{X}$ Type Charts

This section provides some discussions on the $\bar{X}$, synthetic $\bar{X}$, VSI $\bar{X}$ and EWMA $\bar{X}$ charts. These charts are compared with the proposed VSI synthetic $\bar{X}$ chart in Section 5.

### 2.1 The $\bar{X}$ chart

The $\bar{X}$ chart comprises three parallel lines, i.e. the center line (*CL*), lower control limit (*LCL*) and upper control limit (*UCL*). The *CL* represents the target value of the process mean. Assume that a quality characteristic follows a normal distribution with mean *μ*_0_ and standard deviation *σ*, where both *μ*_0_ and *σ* are known. The limits of the $\bar{X}$ chart are

$$LCL=\mu_{0}-k\frac{\sigma }{\sqrt{n}}$$ (1A)

and

$$UCL=\mu_{0}+k\frac{\sigma }{\sqrt{n}},$$ (1B)

where *k* is the width constant that is usually set as 3 so that a Type-I error size of 0.0027 is attained. An out-of-control is issued when the sample mean $\bar{X}$ plots beyond the limit in Eq (1A) or (1B). The *ARL* of the $\bar{X}$ chart which represents the average number of sample points required by the chart to signal a shift in the mean from *μ*_0_ to *μ*_0_ ± *δσ* is calculated as follows:

$$ARL_{\bar{X}}=\frac{1}{q},$$ (2)

where

$$q=1-\Phi \left(k-\delta \sqrt{n}\right)+\Phi \left(-k-\delta \sqrt{n}\right).$$ (3)

Another performance measure is the *ATS*. Here, *ATS* refers to the average number of time periods until a signal is generated by the chart. As the $\bar{X}$ chart involves taking samples at a FSI, its *ATS* is computed as

$$ATS_{\bar{X}}=ARL_{\bar{X}}\times \text{FSI}.$$ (4)

### 2.2 The synthetic $\bar{X}$ chart

**Fig 1:**
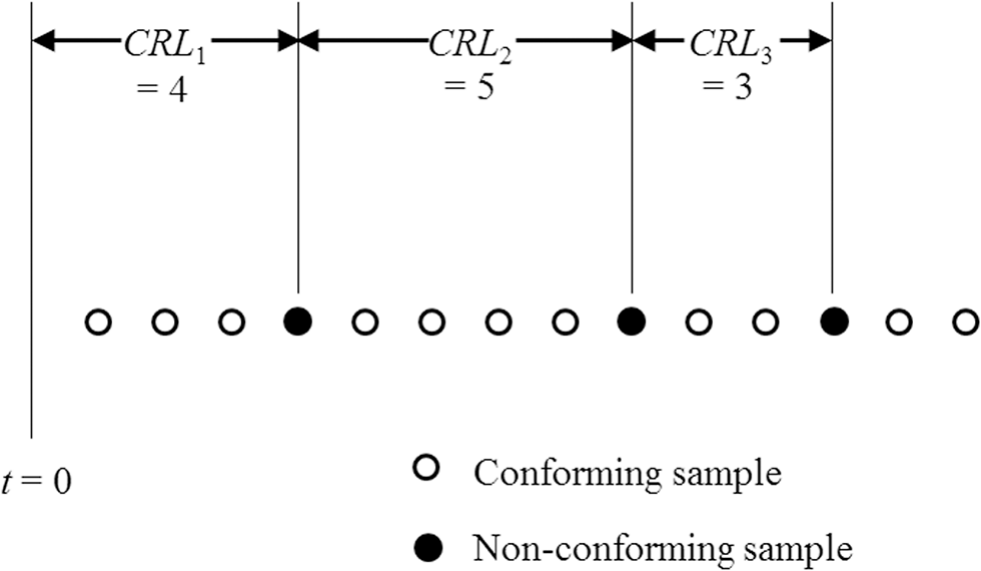
Conforming run length. The CRL value represents the number of inspected samples between two consecutive non-conforming samples in the CRL chart. The conforming and non-conforming samples are represented by the hollow and solid dots on the CRL chart, respectively.

The synthetic $\bar{X}$ chart integrates the Shewhart $\bar{X}$ and *CRL* charts. It comprises the $\bar{X}$/*S* sub-chart and the *CRL*/*S* sub-chart. In the synthetic $\bar{X}$ chart, the *CRL* value refers to the number of inspected samples between two consecutive non-conforming samples, inclusive of the ending nonconforming sample. Fig 1 shows a process starting at *t* = 0 having three *CRL* samples, where *CRL*_1_ = 4, *CRL*_2_ = 5 and *CRL*_3_ = 3. The hollow and solid dots denote the conforming and non-conforming samples, respectively.

The steps for constructing and implementing the synthetic $\bar{X}$ chart are as follows [13]:

1. Determine the lower control limit $LCL_{\bar{X}/S}$ and upper control limit $UCL_{\bar{X}/S}$ of the $\bar{X}$/*S* sub-chart and the lower control limit, *L*_2_ of the *CRL*/*S* sub-chart. $LCL_{\bar{X}/S}$ and $UCL_{\bar{X}/S}$ are computed as
  $$LCL_{\bar{X}/S}=\mu_{0}-k\sigma_{\bar{X}}$$ (5A)
  and
  $$UCL_{\bar{X}/S}=\mu_{0}+k\sigma_{\bar{X}},$$ (5B)
  where *μ*_0_ and $\sigma_{\bar{X}}$ are the in-control mean and standard deviation of the sample mean, respectively. Wu and Spedding [13] presented a procedure to compute the optimal values of *k* and *L*_2_ by minimizing the *ARL*_1_ for a desired size of a standardized mean shift, *δ*_opt_, based on a predefined in-control *ARL* (*ARL*_0_) value.
2. At each inspection point, a random sample of size *n* is taken and the sample mean, $\bar{X}$ is calculated.
3. If the value of $\bar{X}$ falls between the lower control limit $LCL_{\bar{X}/S}$ and upper control limit $UCL_{\bar{X}/S}$, the sample is conforming and the control flow returns to Step (2). Otherwise, the sample is non-conforming and the control flow goes to Step (4).
4. Count the number of $\bar{X}$ samples between the current and the last non-conforming samples (see Fig 1). This number is the *CRL* value of the *CRL*/*S* sub-chart in the synthetic chart.
5. If the *CRL* value is greater than *L*_2_, an in-control status is concluded and the control flow returns to Step (2). Otherwise, an out-of-control status is signalled and the control flow goes to Step (6).
6. Take actions to investigate and eliminate the assignable cause(s). Then return to Step (2).

By assuming that the underlying process follows a normal distribution, the *ARL* formula for the synthetic $\bar{X}$ chart of Wu and Spedding [13] is given as

$$ARL_{\text{Synthetic}\;\bar{X}}=ARL_{\bar{X}}\times ARL_{CRL}=\frac{1}{q}\times \frac{1}{1-{\left(1-q\right)}^{L_{2}}},$$ (6)

where *q* is defined in Eq (3) and *L*_2_ is the lower control limit of the *CRL* sub-chart. The *ATS* formula for the synthetic $\bar{X}$ chart is

$$ATS_{\text{Synthetic}\;\bar{X}}=ARL_{\text{Synthetic}\;\bar{X}}\times \text{FSI}.$$ (7)

### 2.3 The VSI $\bar{X}$ chart

The $\bar{X}$ chart with VSI consists of two sets of limits, i.e. the ±3σ control limits and the warning limits, where the warning limits are located between the in-control mean value and the control limits [19]. When a sample point falls between the warning and control limits, the subsequent sample point may fall outside the control limits with a high chance. Thus, the next sample should be taken as soon as possible (short sampling interval) in order to have a quick detection of changes in the process mean. On the other hand, when a sample point falls between the in-control mean value and warning limits, there is a higher chance for the process to be in-control. Thus, it is reasonable to wait longer (long sampling interval) than the usual time to take the next sample.

Assume that the VSI $\bar{X}$ chart uses a finite number of sampling interval lengths, denoted as *d*_1_,*d*_2_,…,*d*_*m*_, where *d*_1_ < *d*_2_ < ⋯ < *d*_*m*_ and *m* ≥ 2. The choice of a sampling interval can be interpreted as a function of ${\bar{X}}_{i}$ to be $d\left({\bar{x}}_{i}\right)$. Let the interval between the two control limits be partitioned into *I*_1_,*I*_2_,…,*I*_*m*_ sub-intervals, such that

$$d\left({\bar{x}}_{i}\right)=d_{j}\;\text{if}\;{\bar{x}}_{i}\in I_{j},\text{for}\;j=1,2,\ldots ,m.$$ (8)

**Fig 2:**
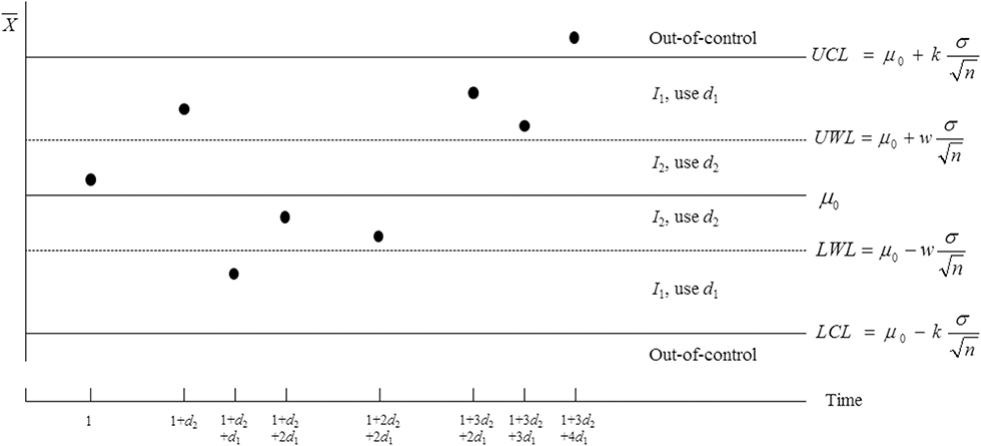
The VSI $\bar{X}$ chart when *m* = 2. The VSI $\bar{X}$ chart is illustrated when two sampling intervals are considered in a process. These sampling intervals are known as the short and long sampling intervals.

Therefore, the sampling interval between samples ${\bar{X}}_{i}$ and ${\bar{X}}_{i+1}$ is $d\left({\bar{x}}_{i}\right)$. Fig 2 illustrates an example of a VSI $\bar{X}$ chart that uses two interval lengths, *d*_1_ and *d*_2_, having

$$I_{1}=\left(\mu_{0}-k\frac{\sigma }{\sqrt{n}},\mu_{0}-w\frac{\sigma }{\sqrt{n}}\right)\cup \left(\mu_{0}+w\frac{\sigma }{\sqrt{n}},\mu_{0}+k\frac{\sigma }{\sqrt{n}}\right)$$ (9)

and

$$I_{2}=\left(\mu_{0}-w\frac{\sigma }{\sqrt{n}},\mu_{0}+w\frac{\sigma }{\sqrt{n}}\right),$$ (10)

where 0 < *w* < *k* and 0 < *d*_1_ < *d*_2_. The VSI $\bar{X}$ chart in Fig 2 is constructed by plotting the sample means against the time on the horizontal axis. When the sample mean falls in *I*_2_, the long sampling interval *d*_2_ is used to take the next sample. However, when the sample mean falls in *I*_1_, the short sampling interval *d*_1_ is adopted to take the next sample. Note that the underlying process is assumed to follow a normal distribution with mean *μ*_0_ and standard deviation *σ*.

Let *p*_1_ and *p*_2_ be the following probabilities (see Fig 2):

$$\begin{array}{l} p_{1}=\text{Pr}\left(UWL<\bar{X}<UCL\right)+\text{Pr}\left(LCL<\bar{X}<LWL\right)\\ \;=\Phi \left(k-\delta \sqrt{n}\right)-\Phi \left(w-\delta \sqrt{n}\right)+\Phi \left(-w-\delta \sqrt{n}\right)-\Phi \left(-k-\delta \sqrt{n}\right) \end{array}$$ (11)

and

$$\begin{array}{l} p_{2}=\text{Pr}\left(LWL<\bar{X}<UWL\right)\\ \;=\Phi \left(w-\delta \sqrt{n}\right)-\Phi \left(-w-\delta \sqrt{n}\right), \end{array}$$ (12)

where *LWL* and *UWL* represent the lower and upper warning limits of the VSI $\bar{X}$ chart, respectively. By using the definition of *q* in Eq (3), we have *p*_1_ + *p*_2_ = 1 − *q*. The average sampling interval, $E\left(T_{\bar{X}}\right)$ of the VSI $\bar{X}$ chart is evaluated as

$$E\left(T_{\bar{X}}\right)=\frac{d_{1}p_{1}+d_{2}p_{2}}{1-q}.$$ (13)

Then the *ATS* formula of the chart is

$$ATS_{\text{VSI}\;\bar{X}}=ARL_{\bar{X}}\times E\left(T_{\bar{X}}\right)=\frac{d_{1}p_{1}+d_{2}p_{2}}{q(1-q)}.$$ (14)

### 2.4 The EWMA $\bar{X}$ chart

The EWMA $\bar{X}$ chart’s statistic, *Z*_*i*_ is given as follows:

$$Z_{i}=\lambda {\bar{X}}_{i}+(1-\lambda )Z_{i-1},\;\text{for}\;i=1,2,\ldots ,$$ (15)

where ${\bar{X}}_{i}$ is the *i* ^th^ sample mean and *Z*_0_ = *μ*_0_. The control limits of the chart are

$$\mu_{0}\pm K\sigma ,$$ (16)

where $K=h\sqrt{\lambda /(n(2-\lambda ))}$ with the multiplier *h* to be decided. The Markov chain approach presented in [31] is used to evaluate the *ARL* of the EWMA $\bar{X}$ chart. The optimal parameters (*λ*,*K*) of the EWMA $\bar{X}$ chart are obtained from the *ARL* criterion to provide the smallest *ARL* for a specified size of shift in the mean when the *ARL*_0_ is set at a desired value. Then the *ATS* formula for the EWMA $\bar{X}$ chart is computed as

$$ATS_{\text{EWMA}\;\bar{X}}=ARL_{\text{EWMA}\;\bar{X}}\times \text{FSI}.$$ (17)

## The VSI Synthetic $\bar{X}$ Chart

### 3.1. Description of the VSI synthetic $\bar{X}$ chart

The fundamental concept of the VSI feature is that the sampling interval for taking the next sample should be short (shorter than the usual length of the sampling interval used for the FSI feature) if the current sample reveals a potential change in the process. However, the sampling interval for taking the next sample should be long (longer than the usual length of the sampling interval used for the FSI feature) if the current sample shows no tendency of a change in the process. In this section, the above concept is used to implement the VSI feature on the synthetic $\bar{X}$ chart, where the $\bar{X}$ and *CRL* sub-charts, each either uses the short or long sampling interval.

Let $T_{\bar{X}}$ represent the sampling interval for taking the $\bar{X}$ samples in the VSI $\bar{X}$ sub-chart, where the length is determined by the location of the $\bar{X}$ sample on the VSI $\bar{X}$ sub-chart as follows:

$$T_{\bar{X}}=\left\{ \begin{array}{l} d_{1},\;\text{if}\;LCL<\bar{X}<LWL\;\text{or}\;UWL<\bar{X}<UCL\\ d_{2},\;\text{if}\;LWL<\bar{X}<UWL \end{array}\right..$$ (18)

**Fig 3:**
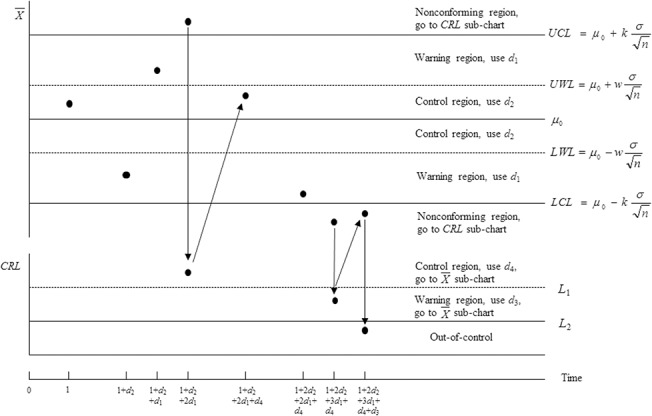
A graphical illustration of the VSI synthetic $\bar{X}$ chart. The VSI synthetic $\bar{X}$ chart consists of the $\bar{X}$ sub-chart and the CRL sub-chart. The short and long sampling intervals are used in the $\bar{X}$ sub-chart and the CRL sub-chart.

Here, *d*_1_ and *d*_2_ represent the length of the short and long sampling intervals, respectively. Assume that the length of the FSI synthetic $\bar{X}$ chart is equal to 1, then *d*_1_ < 1 < *d*_2_. Note that *LCL* and *UCL* represent the lower and upper control limits of the VSI $\bar{X}$ sub-chart, respectively; while *LWL* and *UWL* represent the lower and upper warning limits of the VSI $\bar{X}$ sub-chart, respectively (see Fig 3).

Let *T*_*CRL*_ represent the sampling interval for taking the $\bar{X}$ samples in the VSI $\bar{X}$ sub-chart, where the length is determined by the location of the *CRL* sample on the VSI *CRL* sub-chart as follows:

$$T_{CRL}=\left\{ \begin{array}{l} d_{3}\;\text{if}\;L_{2}<CRL\leq L_{1}\\ d_{4},\;\text{if}\;CRL>L_{1} \end{array}\right..$$ (19)

Here, *d*_3_ and *d*_4_ represent the length of the short and long sampling intervals, respectively, where *d*_3_ < 1 < *d*_4_. *L*_2_ represents the lower control limit of the *CRL* sub-chart while *L*_1_ represents the lower warning limit of the *CRL* sub-chart. From Eqs (18) and (19), three different schemes with the VSI feature for the VSI synthetic $\bar{X}$ chart can be implemented as follows:

(a) VSI $\bar{X}$ sub-chart and FSI *CRL* sub-chart

(fix *d*_3_ = *d*_4_ = 1 but vary *d*_1_ and *d*_2_)

(b) FSI $\bar{X}$ sub-chart and VSI *CRL* sub-chart

(fix *d*_1_ = *d*_2_ = 1 but vary *d*_3_ and *d*_4_)

(c) VSI $\bar{X}$ sub-chart and VSI *CRL* sub-chart

(vary *d*_1_,*d*_2_,*d*_3_ and *d*_4_)

This paper considers varying the sampling intervals for taking the $\bar{X}$ samples based on the information from both the $\bar{X}$ and *CRL* sub-charts (scheme (c)) to provide greater flexibility in the design of the VSI synthetic $\bar{X}$ chart. Fig 3 provides a graphical illustration of the VSI $\bar{X}$ and VSI *CRL* sub-charts of the VSI synthetic $\bar{X}$ chart. Note that when the $\bar{X}$ sample falls in the nonconforming region ($\bar{X}$ < *LCL* or $\bar{X}$ > *UCL*) of the VSI $\bar{X}$ sub-chart, the *CRL* sample is plotted simultaneously on the *CRL* sub-chart, and the sampling interval to take the next $\bar{X}$ sample depends on the location of this *CRL* sample on the *CRL* sub-chart. When the *CRL* sample falls in the out-of-control region (*CRL* ≤ *L*_2_) of the *CRL* sub-chart, the VSI synthetic $\bar{X}$ chart will issue an out-of-control signal.

The operation of the VSI synthetic $\bar{X}$ chart is as follows:

1. Decide on the short and long sampling intervals, *d*_1_, *d*_2_, *d*_3_ and *d*_4_ for the VSI $\bar{X}$ and VSI *CRL* sub-charts, and determine the sample size *n*.
2. Compute the control and warning limits of the VSI $\bar{X}$ and VSI *CRL* sub-charts, i.e. *LCL*, *LWL*, *UWL*, *UCL*, *L*_1_ and *L*_2_. The control and warning limits of the VSI $\bar{X}$ sub-chart are
  $$LCL=\mu_{0}-k\frac{\sigma }{\sqrt{n}},$$ (20A)
  $$LWL=\mu_{0}-w\frac{\sigma }{\sqrt{n}},$$ (20B)
  $$UWL=\mu_{0}+w\frac{\sigma }{\sqrt{n}}$$ (20C)
  and
  $$UCL=\mu_{0}+k\frac{\sigma }{\sqrt{n}},$$ (20D)
  where *μ*_0_ and *σ* are the in-control process mean and standard deviation of the underlying distribution, respectively. The control limits coefficient, *k* is usually larger than the warning limits coefficient, *w*. The computation of the optimal values of *k* and *w* will be discussed in Section 4. Section 4 also explains the computation of the optimal warning and control limits of the *CRL* sub-chart, i.e. *L*_1_ and *L*_2_, respectively.
3. Take a sample of size *n*, then compute the sample mean $\bar{X}$ and plot its value on the $\bar{X}$ sub-chart.
4. If $LWL<\bar{X}<UWL$, the next $\bar{X}$ sample will be taken (and plotted on the VSI $\bar{X}$ sub-chart) after the long sampling interval, *d*_2_. If $LCL<\bar{X}<LWL$ or $UWL<\bar{X}<UCL$, the next $\bar{X}$ sample will be taken (and plotted on the VSI $\bar{X}$ sub-chart) after the short sampling interval, *d*_1_. If $LCL<\bar{X}<UCL$, the $\bar{X}$ sample is conforming and the control flow goes back to Step (3). However, if $\bar{X}$ > *UCL* or $\bar{X}$ < *LCL*, the sample is nonconforming and the control flow proceeds to Step (5).
5. Compute the *CRL* and plot its value on the *CRL* sub-chart.
6. If *CRL* > *L*_1_, the next $\bar{X}$ sample is obtained (and plotted on the VSI $\bar{X}$ sub-chart) after the long sampling interval, *d*_4_. If *L*_2_ < *CRL* ≤ *L*_1_, take the next $\bar{X}$ sample (and plot its value on the VSI $\bar{X}$ sub-chart) after the short sampling interval, *d*_3_. If *CRL* > *L*_2_, the process is in-control and return to Step (3), otherwise, go to Step (7).
7. The VSI synthetic $\bar{X}$ chart signals an out-of-control.
8. Investigate the process for the presence of assignable cause(s) and make process adjustments so that the out-of-control process is brought back into an in-control condition.
9. Once the process returns to the in-control condition, go back to Step (3) and continue with process monitoring.

Note that the VSI synthetic $\bar{X}$ chart does not trigger an out-of-control condition when the $\bar{X}$ sample falls beyond the *UCL/LCL* limits of the VSI $\bar{X}$ sub-chart. An out-of-control is only signalled by the VSI synthetic $\bar{X}$ chart when the *CRL* value is smaller than or equal to *L*_2_.

### 3.2 Properties of the VSI synthetic $\bar{X}$ chart

The *ARL* formula of the synthetic $\bar{X}$ chart in Eq (6) can be rearranged as follows [30]:

$$ARL=1+[ARL_{\bar{X}}-1](ARL_{CRL})+ARL_{CRL}-1,$$ (21)

where the formulae for computing $ARL_{\bar{X}}$ and *ARL*_*CRL*_ are shown in Eq (6). The rearranged *ARL* formula can be divided into 3 parts. The first part “1” is the initial sampling interval. The second part “$\left[ARL_{\bar{X}}-1](ARL_{CRL}\right)$” is the expected number of sampling intervals, where the length is determined by the $\bar{X}$ samples, while the last part “*ARL*_*CRL*_–1” is the expected number of sampling intervals, where the length is dependent on the *CRL* samples. When the VSI feature is considered, *ATS* is used to measure the performance of the VSI synthetic $\bar{X}$ chart, where the sampling intervals of the VSI $\bar{X}$ and VSI *CRL* sub-charts are allowed to vary. Several sampling intervals can be used to measure the performance of the chart; however, this research just considers two sampling intervals which are the short and long sampling intervals. The long sampling interval is taken when the sample point is located in the control region and the short sampling interval is used when the sample point falls in the warning region. When the lengths of the sampling intervals are considered, the basic synthetic $\bar{X}$ chart becomes the VSI synthetic $\bar{X}$ chart, and the *ARL* in Eq (21) becomes the following *ATS*:

$$ATS=t_{f}+[ARL_{\bar{X}}-1](ARL_{CRL})[E(T_{\bar{X}})]+[ARL_{CRL}-1][E(T_{CRL})],$$ (22)

where

$$\begin{array}{l} E(T_{\bar{X}})=\frac{d_{1}[\text{Pr}(UWL<\bar{X}<UCL)+\text{Pr}(LCL<\bar{X}<LWL)]+d_{2}[\text{Pr}(LWL<\bar{X}<UWL)]}{\left(1-q\right)}\\ =\frac{d_{1}\left[\Phi \left(k-\delta \sqrt{n}\right)-\Phi \left(w-\delta \sqrt{n}\right)+\Phi \left(-w-\delta \sqrt{n}\right)-\Phi \left(-k-\delta \sqrt{n}\right)\right]+d_{2}\left[\Phi \left(w-\delta \sqrt{n}\right)-\Phi \left(-w-\delta \sqrt{n}\right)\right]}{1-\Phi \left(k-\delta \sqrt{n}\right)+\Phi \left(-k-\delta \sqrt{n}\right)} \end{array}$$ (23)

and

$$\begin{array}{l} E(T_{CRL})=\frac{d_{3}\;\text{Pr}(L_{2}<CRL\leq L_{1})+d_{4}\;\text{Pr}(CRL>L_{1})}{\text{Pr}(CRL>L_{2})}\\ \;=\frac{d_{3}\left[{\left(1-q\right)}^{L_{2}}-{\left(1-q\right)}^{L_{1}}\right]+d_{4}{\left(1-q\right)}^{L_{1}}}{{\left(1-q\right)}^{L_{2}}}. \end{array}$$ (24)

Note that $E\left(T_{\bar{X}}\right)$ and *E*(*T*_*CRL*_) are the expected value of $T_{\bar{X}}$ and *T*_*CRL*_, respectively and the *ATS* formula in Eq (22) is used as a performance criterion for the VSI synthetic $\bar{X}$ chart.

## Optimal Design of the VSI Synthetic $\bar{X}$ Chart

In statistical design, an optimal selection of the parameters, *k*, *w*, *L*_1_ and *L*_2_ for the VSI synthetic $\bar{X}$ chart is important to minimize the *ATS*_1_ for a desired size of a mean shift. When the process is in-control, the VSI synthetic $\bar{X}$ chart is designed to have the same false alarm rate as the basic synthetic $\bar{X}$ chart. For this reason, the in-control *ATS* (*ATS*_0_) of the VSI synthetic $\bar{X}$ chart is set to be equal to *ARL*_0_×FSI of the basic synthetic $\bar{X}$ chart. The FSI of the basic synthetic $\bar{X}$ chart is usually set as unity so that the chart's *ATS*_0_ and *ARL*_0_ are similar. Hence, to ensure that *ATS*_0_ of the VSI synthetic $\bar{X}$ chart is similar to that of the basic synthetic $\bar{X}$ chart, $E\left(T_{\bar{X}}\right)$, *E*(*T*_*CRL*_) and *t*_*f*_ in Eq (22) should be set as unity when the process is in-control.

The optimal design procedure for the VSI synthetic $\bar{X}$ chart to minimize the *ATS*_1_ is described in the following steps:

1. Fix *d*_1_, *d*_2_ and *d*_3_ (for instance, fix *d*_1_ and *d*_3_ as 0.5, and *d*_2_ as 1.5). Then set $E(T_{\bar{X}})=E(T_{CRL})=1$ and *t*_*f*_ = 1, when the process is in-control so that *ATS*_0_ of the VSI synthetic $\bar{X}$ chart (see Eq (22)) becomes the *ARL*_0_ (or *ATS*_0_ when FSI = 1) of the synthetic $\bar{X}$ chart (see Eq (21)).
2. Specify the nominal *ATS*_0_ and set the sample size, *n*.
3. Choose the magnitude of the standardized mean shift, δ_opt_, where a quick detection is needed.
4. Determine the optimal parameters *k* and *L*_2_ of the $\bar{X}$ and *CRL* sub-charts, respectively, for the synthetic $\bar{X}$ chart, based on the optimization procedure recommended by Wu and Spedding [13]. Note that the VSI synthetic $\bar{X}$ chart is designed to have the same limits constants, *k* and *L*_2_ as that of the synthetic $\bar{X}$ chart.
5. Once the value of the optimal parameter *k* is determined, the value of parameter *w* can be determined from the $E\left(T_{\bar{X}}\right)$ formula in Eq (23), as $E\left(T_{\bar{X}}\right)$ is set as unity in Step (1) when the process is in-control. Then initialize *L*_1_ as *L*_1_ = *L*_2_ + 1.
6. Determine *d*_4_ using Eq (24) when the process is in-control (δ = 0) as *E*(*T*_*CRL*_) is set as unity (see Step (1)).
7. Compute *ATS*_1_ when δ = δ_opt_, denoted as *ATS*_1_(δ_opt_). If *L*_1_ = *L*_2_ + 1, increase *L*_1_ by one and go to Step (6); otherwise go to Step (8).
8. If *ATS*_1_(δ_opt_) has been reduced, increase *L*_1_ by one and return to Step (6). Otherwise, proceed to Step (9).
9. Record the smallest *ATS*_1_(δ_opt_) and take the corresponding *k*, *w*, *L*_1_, *L*_2_, *d*_1_, *d*_2_, *d*_3_ and *d*_4_ values as the optimal parameters of the chart.

The optimal parameters (*L*_1_,*L*_2_,*k*,*w*,*d*_4_) of the VSI synthetic $\bar{X}$ chart, for *d*_1_, *d*_2_ and *d*_3_ = 0.5, 1.5 and 0.5, respectively, based on *n* = 3, 5, 7 and 9, and *ATS*_0_ = 370 are presented in Table 1. These optimal parameters ensure that the VSI synthetic $\bar{X}$ chart gives the smallest *ATS*_1_ value for the standardized mean shift δ_opt_ of interest shown in Table 1. Note that δ_opt_ is the size of a mean shift for which a quick detection is needed. These optimal parameters are computed using an optimization program written in the ScicosLab software. This program can be requested from the first author. The accuracies of the results in Table 1 have been verified with simulation.

**Table 1:** Optimal parameters (*L*_1_,*L*_2_,*k*,*w*,*d*_4_) and the corresponding *ATS*_1_(δ_opt_) for VSI synthetic $\bar{X}$ chart, for sample sizes, *n* = 3, 5, 7 and 9, and *ATS*_0_ = 370 when *d*_1_, *d*_2_ and *d*_3_ are set as 0.5, 1.5 and 0.5, respectively.

δ_opt_	*n* = 3						*n* = 5						*n* = 7						*n* = 9					
	*L*_1_	*L*_2_	*k*	*w*	*d*_4_	*ATS*_1_(δ_opt_)	*L*_1_	*L*_2_	*k*	*w*	*d*_4_	*ATS*_1_(δ_opt_)	*L*_1_	*L*_2_	*k*	*w*	*d*_4_	*ATS*_1_(δ_opt_)	*L*_1_	*L*_2_	*k*	*w*	*d*_4_	*ATS*_1_(δ_opt_)
0.1	29965	103	2.75	0.67	4.1×10^76^	298.94	17806	95	2.74	0.67	8×10^46^	262.74	12908	89	2.73	0.67	6.7×10^34^	232.83	9851	83	2.72	0.67	1.9×10^27^	207.80
0.2	7175	75	2.71	0.67	4.4×10^20^	177.26	4091	60	2.68	0.67	6.5×10^12^	122.45	2729	50	2.65	0.67	1.4×10^9^	89.66	1979	42	2.62	0.67	1.2×10^7^	68.47
0.3	2850	51	2.65	0.67	3.1×10^9^	92.99	1517	36	2.60	0.67	582673.04	52.49	971	28	2.56	0.67	10857.71	33.64	698	22	2.52	0.67	1505.24	23.38
0.4	1393	34	2.59	0.67	259093.10	48.39	713	23	2.53	0.67	1501.67	23.82	452	17	2.48	0.66	168.79	14.18	311	13	2.43	0.66	46.78	9.48
0.5	782	24	2.53	0.67	2779.96	26.20	385	15	2.45	0.66	95.95	11.90	238	11	2.40	0.66	21.46	6.92	163	8	2.35	0.66	10.28	4.65
0.6	471	17	2.48	0.66	217.49	15.02	232	11	2.40	0.66	19.49	6.61	143	8	2.35	0.66	7.16	3.91	98	6	2.29	0.66	4.30	2.73
0.7	304	13	2.43	0.66	42.11	9.17	146	8	2.35	0.66	7.56	4.08	91	6	2.29	0.66	3.76	2.54	61	4	2.22	0.65	2.81	1.89
0.8	208	10	2.38	0.66	15.65	5.96	98	6	2.29	0.66	4.30	2.78	60	4	2.22	0.65	2.75	1.85	42	3	2.16	0.65	2.17	1.47
0.9	147	8	2.35	0.66	7.69	4.13	71	5	2.26	0.66	2.95	2.07	42	3	2.16	0.65	2.17	1.49	30	3	2.16	0.65	1.65	1.26
1	107	6	2.29	0.66	5.14	3.04	52	4	2.22	0.65	2.32	1.66	32	3	2.16	0.65	1.73	1.29	22	2	2.08	0.65	1.57	1.14
1.1	81	5	2.26	0.66	3.62	2.36	39	3	2.16	0.65	2.02	1.42	25	3	2.16	0.65	1.49	1.17	17	2	2.08	0.65	1.38	1.07
1.2	63	4	2.22	0.65	2.94	1.93	31	3	2.16	0.65	1.69	1.27	19	2	2.08	0.65	1.45	1.10	14	2	2.08	0.65	1.29	1.04
1.3	52	4	2.22	0.65	2.32	1.64	25	3	2.16	0.65	1.49	1.17	15	2	2.08	0.65	1.32	1.06	11	2	2.08	0.65	1.20	1.02
1.4	40	3	2.16	0.65	2.07	1.45	20	2	2.08	0.65	1.49	1.11	13	2	2.08	0.65	1.26	1.03	9	2	2.08	0.65	1.15	1.01
1.5	33	3	2.16	0.65	1.77	1.32	17	2	2.08	0.65	1.38	1.07	11	2	2.08	0.65	1.20	1.02	8	2	2.08	0.65	1.13	1.00
1.6	28	3	2.16	0.65	1.58	1.22	14	2	2.08	0.65	1.29	1.04	9	2	2.08	0.65	1.15	1.01	8	2	2.08	0.65	1.13	1.00
1.7	23	2	2.08	0.65	1.61	1.16	12	2	2.08	0.65	1.23	1.02	8	2	2.08	0.65	1.13	1.00	6	2	2.08	0.65	1.08	1.00
1.8	20	2	2.08	0.65	1.49	1.11	10	2	2.08	0.65	1.18	1.01	7	2	2.08	0.65	1.10	1.00	5	2	2.08	0.65	1.06	1.00
1.9	17	2	2.08	0.65	1.38	1.08	9	2	2.08	0.65	1.15	1.01	6	2	2.08	0.65	1.08	1.00	5	2	2.08	0.65	1.06	1.00
2	15	2	2.08	0.65	1.32	1.05	8	2	2.08	0.65	1.13	1.00	5	2	2.08	0.65	1.06	1.00	4	2	2.08	0.65	1.04	1.00
2.2	12	2	2.08	0.65	1.23	1.02	6	2	2.08	0.65	1.08	1.00	5	2	2.08	0.65	1.06	1.00	4	2	2.08	0.65	1.04	1.00
2.4	10	2	2.08	0.65	1.18	1.01	6	2	2.08	0.65	1.08	1.00	4	2	2.08	0.65	1.04	1.00	3	1	1.94	0.63	1.06	1.00
2.6	8	2	2.08	0.65	1.13	1.00	4	2	2.08	0.65	1.04	1.00	3	1	1.94	0.63	1.06	1.00	2	1	1.94	0.63	1.03	1.00
2.8	7	2	2.08	0.65	1.10	1.00	4	2	2.08	0.65	1.04	1.00	3	1	1.94	0.63	1.06	1.00	2	1	1.94	0.63	1.03	1.00
3	6	2	2.08	0.65	1.08	1.00	3	2	2.08	0.65	1.02	1.00	3	1	1.94	0.63	1.06	1.00	2	1	1.94	0.63	1.03	1.00

The *ATS*_0_ is set as 370 so that the in-control performance of the VSI synthetic $\bar{X}$ chart match that of a typical 3 sigma Shewhart $\bar{X}$ chart. With this condition, the detection effectiveness of the proposed chart can be compared with other charts which are designed under the same set of specifications. In Table 1, when the mean shift δ_opt_ increases, the *ATS*_1_(δ_opt_) value decreases towards unity. Furthermore, as the sample size, *n* increases from *n* = 3 to *n* = 9, the *ATS*_1_(δ_opt_) value decreases towards unity quicker as δ_opt_ increases. For example, *ATS*_1_(δ_opt_) reaches unity when δ_opt_ = 2.6, 2, 1.7 and 1.5 for *n* = 3, 5, 7 and 9, respectively. This indicates that the VSI synthetic $\bar{X}$ chart provides a quicker detection of shifts in the mean when the sample size, *n* increases. Note that when δ_opt_ is greater than 0.1, all the *ATS*_1_(δ_opt_) values are less than half of the *ATS*_0_ value.

## Average Time to Signal (ATS) Comparisons

In this section, four $\bar{X}$ type control charts, namely, the $\bar{X}$, synthetic $\bar{X}$, VSI $\bar{X}$ and EWMA $\bar{X}$ charts are compared with the VSI synthetic $\bar{X}$ chart, based on the *ATS* performance. All the charts, except the $\bar{X}$ chart, are designed to minimize *ATS*_1_(δ_opt_). Note that δ_opt_ is a standardized mean shift, where a quick detection is needed. *ATS*_0_ is set as 370 for all the charts when the sample sizes, *n* = 3, 5, 7 and 9, optimal shifts, δ_opt_ = {0.1, 0.2, …, 2.0, 2.2, …, 3.0} so that a fair comparison can be made. The FSI of the $\bar{X}$, synthetic $\bar{X}$ and EWMA $\bar{X}$ charts is set as unity so that the *ATS* values of these charts are the same as their ARL values.

The initial sampling interval, *t*_*f*_, for the VSI $\bar{X}$ and VSI synthetic $\bar{X}$ charts is set as unity so that these two charts start with the same sampling interval length. The lengths of the short and long sampling intervals decided by the $\bar{X}$ sub-chart of both the VSI $\bar{X}$ and VSI synthetic $\bar{X}$ charts are set as 0.5 hour and 1.5 hours (*d*_1_ = 0.5 and *d*_2_ = 1.5), respectively. In addition, the length of the short sampling interval decided by the *CRL* sub-chart of the VSI synthetic $\bar{X}$ chart is set as 0.5 hour (*d*_3_ = 0.5) while the corresponding long sampling interval (*d*_4_) is computed by letting *E*(*T*_*CRL*_) = 1, as mentioned in Section 4.

The optimal parameters for the synthetic $\bar{X}$, VSI $\bar{X}$ and EWMA $\bar{X}$ charts are given in Table 2 while that for the VSI synthetic $\bar{X}$ chart are shown in Table 1. The procedures to compute the optimal parameters by minimizing *ATS*_1_(δ_opt_) of these charts (or equivalently *ARL*_1_(δ_opt_) of the synthetic $\bar{X}$ and EWMA $\bar{X}$ charts) are explained in [13,19,31] for the synthetic $\bar{X}$, VSI $\bar{X}$ and EWMA $\bar{X}$ charts, respectively. Concerning the VSI synthetic $\bar{X}$ chart, its optimal parameters are computed using the approach described in Section 4. The width constant of the $\bar{X}$ chart is set as *k* = 3 as *ATS*_0_ = *ARL*_0_ = 370. The *ATS*_1_(δ_opt_) values for the $\bar{X}$, synthetic $\bar{X}$, VSI $\bar{X}$ and EWMA $\bar{X}$ charts are computed using the formulae given in Section 2 while that for the VSI synthetic $\bar{X}$ chart is obtained using Eq (22).

**Table 2:** Optimal parameters for synthetic $\bar{X}$, VSI $\bar{X}$ and EWMA $\bar{X}$ charts to minimize *ATS*_1_(δ_opt_) when *n* = 3, 5, 7 and 9, and ATS_0_ = 370.

δ_opt_	*n* = 3			*n* = 5			*n* = 7			*n* = 9		
	Synthetic $\bar{X}$ (*L*_2_,*k*)	$\text{VSI}\;\bar{X}$ (*k*, *w*)	EWMA $\bar{X}$ (*λ*, *K*)	Synthetic $\bar{X}$ $\left(L_{2},k\right)$	$\text{VSI}\;\bar{X}$ (*k*, *w*)	EWMA $\bar{X}$ (*λ*, *K*)	Synthetic $\bar{X}$ (*L*_2_,*k*)	$\text{VSI}\;\bar{X}$ (*k*, *w*)	EWMA $\bar{X}$ (*λ*, *K*)	Synthetic $\bar{X}$ (*L*_2_,*k*)	$\text{VSI}\;\bar{X}$ (*k*, *w*)	EWMA $\bar{X}$ (*λ*, *K*)
0.1	(103, 2.753)	(3, 0.672)	(0.031, 0.167)	(95, 2.741)	(3, 0.672)	(0.015, 0.077)	(89, 2.732)	(3, 0.672)	(0.019, 0.079)	(83, 2.722)	(3, 0.672)	(0.022, 0.077)
0.2	(75, 2.708)	(3, 0.672)	(0.031, 0.167)	(60, 2.675)	(3, 0.672)	(0.042, 0.159)	(50, 2.648)	(3, 0.672)	(0.054, 0.159)	(42, 2.622)	(3, 0.672)	(0.066, 0.159)
0.3	(51, 2.651)	(3, 0.672)	(0.053, 0.241)	(36, 2.598)	(3, 0.672)	(0.081, 0.244)	(28, 2.558)	(3, 0.672)	(0.101, 0.236)	(22, 2.519)	(3, 0.672)	(0.122, 0.234)
0.4	(34, 2.589)	(3, 0.672)	(0.082, 0.316)	(23, 2.526)	(3, 0.672)	(0.120, 0.310)	(17, 2.476)	(3, 0.672)	(0.154, 0.306)	(13, 2.430)	(3, 0.672)	(0.185, 0.302)
0.5	(24, 2.533)	(3, 0.672)	(0.114, 0.388)	(15, 2.455)	(3, 0.672)	(0.166, 0.380)	(11, 2.402)	(3, 0.672)	(0.213, 0.375)	(8, 2.346)	(3, 0.672)	(0.256, 0.370)
0.6	(17, 2.476)	(3, 0.672)	(0.150, 0.461)	(11, 2.402)	(3, 0.672)	(0.217, 0.448)	(8, 2.346)	(3, 0.672)	(0.276, 0.440)	(6, 2.294)	(3, 0.672)	(0.330, 0.435)
0.7	(13, 2.430)	(3, 0.672)	(0.187, 0.528)	(8, 2.346)	(3, 0.672)	(0.270, 0.515)	(6, 2.294)	(3, 0.672)	(0.342, 0.505)	(4, 2.219)	(3, 0.672)	(0.411, 0.502)
0.8	(10, 2.385)	(3, 0.672)	(0.227, 0.596)	(6, 2.294)	(3, 0.672)	(0.326, 0.580)	(4, 2.219)	(3, 0.672)	(0.418, 0.576)	(3, 2.164)	(3, 0.672)	(0.510, 0.581)
0.9	(8, 2.346)	(3, 0.672)	(0.269, 0.663)	(5, 2.260)	(3, 0.672)	(0.387, 0.647)	(3, 2.164)	(3, 0.672)	(0.502, 0.652)	(3, 2.164)	(3, 0.672)	(0.608, 0.658)
1	(6, 2.294)	(3, 0.672)	(0.312, 0.727)	(4, 2.219)	(3, 0.672)	(0.455, 0.721)	(3, 2.164)	(3, 0.672)	(0.591, 0.731)	(2, 2.085)	(3, 0.672)	(0.698, 0.731)
1.1	(5, 2.260)	(3, 0.672)	(0.357, 0.793)	(3, 2.164)	(3, 0.672)	(0.529, 0.799)	(3, 2.164)	(3, 0.672)	(0.675, 0.808)	(2, 2.085)	(3, 0.672)	(0.772, 0.792)
1.2	(4, 2.219)	(3, 0.672)	(0.405, 0.861)	(3, 2.164)	(3, 0.672)	(0.603, 0.878)	(2, 2.085)	(3, 0.672)	(0.746, 0.873)	(2, 2.085)	(3, 0.672)	(0.834, 0.845)
1.3	(4, 2.219)	(3, 0.672)	(0.460, 0.938)	(3, 2.164)	(3, 0.672)	(0.672, 0.952)	(2, 2.085)	(3, 0.672)	(0.804, 0.929)	(2, 2.085)	(3, 0.672)	(0.882, 0.888)
1.4	(3, 2.164)	(3, 0.672)	(0.517, 1.015)	(2, 2.085)	(3, 0.672)	(0.732, 1.018)	(2, 2.085)	(3, 0.672)	(0.854, 0.978)	(2, 2.085)	(3, 0.672)	(0.922, 0.924)
1.5	(3, 2.164)	(3, 0.672)	(0.576, 1.097)	(2, 2.085)	(3, 0.672)	(0.783, 1.075)	(2, 2.085)	(3, 0.672)	(0.893, 1.018)	(2, 2.085)	(3, 0.672)	(0.952, 0.953)
1.6	(3, 2.164)	(3, 0.672)	(0.632, 1.173)	(2, 2.085)	(3, 0.672)	(0.830, 1.129)	(2, 2.085)	(3, 0.672)	(0.925, 1.052)	(2, 2.085)	(3, 0.672)	(0.972, 0.972)
1.7	(2, 2.085)	(3, 0.672)	(0.683, 1.245)	(2, 2.085)	(3, 0.672)	(0.868, 1.174)	(2, 2.085)	(3, 0.672)	(0.951, 1.080)	(2, 2.085)	(3, 0.672)	(0.985, 0.985)
1.8	(2, 2.085)	(3, 0.672)	(0.730, 1.311)	(2, 2.085)	(3, 0.672)	(0.902, 1.215)	(2, 2.085)	(3, 0.672)	(0.973, 1.104)	(2, 2.085)	(3, 0.672)	(0.994, 0.994)
1.9	(2, 2.085)	(3, 0.672)	(0.770, 1.369)	(2, 2.085)	(3, 0.672)	(0.928, 1.248)	(2, 2.085)	(3, 0.672)	(0.983, 1.115)	(2, 2.085)	(3, 0.672)	(0.999, 0.999)
2	(2, 2.085)	(3, 0.672)	(0.808, 1.425)	(2, 2.085)	(3, 0.672)	(0.950, 1.276)	(2, 2.085)	(3, 0.672)	(0.988, 1.121)	(2, 2.085)	(3, 0.672)	(0.999, 0.999)
2.2	(2, 2.085)	(3, 0.672)	(0.869, 1.518)	(2, 2.085)	(3, 0.672)	(0.978, 1.313)	(2, 2.085)	(3, 0.672)	(0.999, 1.133)	(2, 2.085)	(3, 0.672)	(0.999, 0.999)
2.4	(2, 2.085)	(3, 0.672)	(0.918, 1.595)	(2, 2.085)	(3, 0.672)	(0.991, 1.330)	(2, 2.085)	(3, 0.672)	(0.999, 1.133)	(1, 1.943)	(3, 0.672)	(0.999, 0.999)
2.6	(2, 2.085)	(3, 0.672)	(0.953, 1.652)	(2, 2.085)	(3, 0.672)	(0.997, 1.338)	(1, 1.943)	(3, 0.672)	(0.999, 1.133)	(1, 1.943)	(3, 0.672)	(0.999, 0.999)
2.8	(2, 2.085)	(3, 0.672)	(0.975, 1.689)	(2, 2.085)	(3, 0.672)	(0.998, 1.340)	(1, 1.943)	(3, 0.672)	(0.999, 1.133)	(1, 1.943)	(3, 0.672)	(0.999, 0.999)
3	(2, 2.085)	(3, 0.672)	(0.988, 1.711)	(2, 2.085)	(3, 0.672)	(0.998, 1.340)	(1, 1.943)	(3, 0.672)	(0.999, 1.133)	(1, 1.943)	(3, 0.672)	(0.999, 0.999)

In Table 2, the optimal parameters (*L*_2_,*k*) of the synthetic $\bar{X}$ chart generally remain constant even though δ_opt_ increases once δ_opt_ is larger than a certain value. In particular, (*L*_2_,*k*) = (2, 2.085) when δ_opt_ is larger than 1.6 and 1.3 for *n* = 3 and 5, respectively. The parameter, *w* of the VSI $\bar{X}$ chart is always equal to 0.672 regardless of the size of δ_opt_ because the width constant of the $\bar{X}$ chart is set as *k* = 3. For the EWMA $\bar{X}$ chart, a larger *λ* corresponds to a larger δ_opt_ value and that *λ* approaches unity when δ_opt_ increases towards 3. This is because when *λ* = 1, the EWMA $\bar{X}$ chart’s statistic reduces to the Shewhart $\bar{X}$ chart’s statistic so that a large shift in the process mean can be detected quicker.

The objective function minimizes the *ATS*_1_(δ_opt_), while at the same time satisfying the *ATS*_0_ requirement. The findings in Table 3 shows that the VSI synthetic $\bar{X}$ chart outperforms the $\bar{X}$, synthetic $\bar{X}$ and VSI $\bar{X}$ charts, for all sizes of shifts, except for very large shifts where all these charts have equal performances (see boldfaced entries in Table 3). For example, when *n* = 9 and δ_opt_ = 0.5 (moderate shift), the *ATS*_1_(δ_opt_) for the VSI synthetic $\bar{X}$ chart is 4.65, while the corresponding *ATS*_1_(δ_opt_) values for the EWMA $\bar{X}$, VSI $\bar{X}$, synthetic $\bar{X}$ and $\bar{X}$ charts are 5.18, 10.81, 6.05 and 14.96, respectively. For this example, the speed to detect a process shift by the VSI synthetic $\bar{X}$ chart is more than two times quicker than the VSI $\bar{X}$ chart and more than three times quicker than the $\bar{X}$ chart. Note that the EWMA $\bar{X}$ chart prevails for detecting small shifts (see boldfaced entries in Table 3) compared with the other $\bar{X}$ type charts but the VSI synthetic $\bar{X}$ chart surpasses the EWMA $\bar{X}$ chart for detecting moderate and large shifts.

**Table 3:** *ATS*_1_(δ_opt_) for the $\bar{X}$, EWMA $\bar{X}$, VSI $\bar{X}$, synthetic $\bar{X}$ and VSI synthetic $\bar{X}$ charts when *n* = 3, 5, 7 and 9, and ATS_0_ = 370.

δ_opt_	*n* = 3					*n* = 5					*n* = 7					*n* = 9				
	$\bar{X}$	EWMA $\bar{X}$	$\text{VSI}\;\bar{X}$	Synthetic $\bar{X}$	VSI Synthetic $\bar{X}$	$\bar{X}$	EWMA $\bar{X}$	$\text{VSI}\;\bar{X}$	Synthetic $\bar{X}$	VSI Synthetic $\bar{X}$	$\bar{X}$	EWMA $\bar{X}$	$\text{VSI}\;\bar{X}$	Synthetic $\bar{X}$	VSI Synthetic $\bar{X}$	$\bar{X}$	EWMA $\bar{X}$	$\text{VSI}\;\bar{X}$	Synthetic $\bar{X}$	VSI Synthetic $\bar{X}$
0.1	321.76	**110.98**	319.77	301.24	298.94	295.45	**76.50**	292.42	265.86	262.74	272.71	**62.12**	268.82	236.55	232.83	252.88	**52.91**	248.26	211.98	207.80
0.2	227.49	**43.82**	221.99	181.92	177.26	177.56	**30.94**	170.54	127.65	122.45	143.79	**24.43**	135.98	94.89	89.66	119.56	**20.40**	111.36	73.52	68.47
0.3	147.40	**25.07**	139.65	98.22	92.99	99.46	**17.35**	91.11	57.25	52.49	72.63	**13.54**	64.44	37.78	33.64	55.78	**11.22**	48.03	26.97	23.38
0.4	93.96	**16.55**	85.61	53.05	48.39	56.55	**11.33**	48.77	27.43	23.82	38.27	**8.79**	31.42	17.01	14.18	27.80	**7.27**	21.86	11.75	9.48
0.5	60.64	**11.89**	52.72	29.97	26.2	33.38	**8.09**	26.91	14.48	11.90	21.37	**6.27**	16.20	8.78	6.92	14.96	5.18	10.81	6.05	**4.65**
0.6	40.00	**9.04**	33.04	17.94	15.02	20.55	**6.13**	15.49	8.42	6.61	12.67	4.75	8.96	5.13	**3.91**	8.69	3.92	5.91	3.60	**2.73**
0.7	27.05	**7.15**	21.19	11.39	9.17	13.21	4.85	9.39	5.34	**4.08**	7.96	3.76	5.38	3.34	**2.54**	5.43	3.11	3.62	2.43	**1.89**
0.8	18.77	**5.84**	13.98	7.65	5.96	8.85	3.96	6.03	3.67	**2.78**	5.30	3.07	3.53	2.38	**1.85**	3.64	2.54	2.47	1.81	**1.47**
0.9	13.37	4.88	9.52	5.41	**4.13**	6.18	3.32	4.13	2.69	**2.07**	3.73	2.57	2.52	1.84	**1.49**	2.62	2.11	1.86	1.46	**1.26**
1	9.76	4.16	6.71	4.01	**3.04**	4.49	2.83	3.00	2.10	**1.66**	2.76	2.18	1.95	1.51	**1.29**	2.00	1.78	1.52	1.26	**1.14**
1.1	7.31	3.60	4.91	3.10	**2.36**	3.39	2.44	2.32	1.72	**1.42**	2.15	1.87	1.60	1.31	**1.17**	1.62	1.53	1.32	1.14	**1.07**
1.2	5.60	3.16	3.73	2.49	**1.93**	2.66	2.13	1.89	1.47	**1.27**	1.76	1.63	1.39	1.18	**1.10**	1.38	1.35	1.19	1.07	**1.04**
1.3	4.40	2.80	2.94	2.06	**1.64**	2.16	1.87	1.61	1.31	**1.17**	1.49	1.44	1.25	1.10	**1.06**	1.23	1.22	1.11	1.04	**1.02**
1.4	3.54	2.50	2.41	1.77	**1.45**	1.81	1.66	1.42	1.20	**1.11**	1.32	1.30	1.16	1.06	**1.03**	1.13	1.13	1.07	1.02	**1.01**
1.5	2.91	2.24	2.03	1.55	**1.32**	1.57	1.49	1.29	1.13	**1.07**	1.20	1.19	1.10	1.03	**1.02**	1.07	1.07	1.04	1.01	**1.00**
1.6	2.44	2.02	1.76	1.40	**1.22**	1.39	1.36	1.20	1.08	**1.04**	1.12	1.12	1.06	1.02	**1.01**	1.04	1.04	1.02	1.00	1.00
1.7	2.09	1.84	1.57	1.29	**1.16**	1.27	1.25	1.14	1.05	**1.02**	1.07	1.07	1.04	1.01	**1.00**	1.02	1.02	1.01	1.00	1.00
1.8	1.83	1.67	1.43	1.20	**1.11**	1.18	1.17	1.09	1.03	**1.01**	1.04	1.04	1.02	1.00	1.00	1.01	1.01	1.00	1.00	1.00
1.9	1.63	1.54	1.32	1.14	**1.08**	1.12	1.12	1.06	1.02	**1.01**	1.02	1.02	1.01	1.00	1.00	1.00	1.00	1.00	1.00	1.00
2	1.47	1.42	1.24	1.10	**1.05**	1.08	1.08	1.04	1.01	**1.00**	1.01	1.01	1.01	1.00	1.00	1.00	1.00	1.00	1.00	1.00
2.2	1.26	1.25	1.13	1.05	**1.02**	1.03	1.03	1.01	1.00	1.00	1.00	1.00	1.00	1.00	1.00	1.00	1.00	1.00	1.00	1.00
2.4	1.14	1.14	1.07	1.02	**1.01**	1.01	1.01	1.00	1.00	1.00	1.00	1.00	1.00	1.00	1.00	1.00	1.00	1.00	1.00	1.00
2.6	1.07	1.07	1.04	1.01	**1.00**	1.00	1.00	1.00	1.00	1.00	1.00	1.00	1.00	1.00	1.00	1.00	1.00	1.00	1.00	1.00
2.8	1.03	1.03	1.02	1.00	1.00	1.00	1.00	1.00	1.00	1.00	1.00	1.00	1.00	1.00	1.00	1.00	1.00	1.00	1.00	1.00
3	1.01	1.01	1.01	1.00	1.00	1.00	1.00	1.00	1.00	1.00	1.00	1.00	1.00	1.00	1.00	1.00	1.00	1.00	1.00	1.00

## An Illustrative Example

In semiconductor manufacturing problem, photolithography is a crucial step in fabrication. The hard-bake process is important in photolithography to increase resist adherence and etch resistance [32]. A critical quality characteristic in the hard-bake process is the flow width of the resist. A dataset taken from [32] is used to illustrate the construction of the VSI synthetic $\bar{X}$ chart for monitoring the process mean of flow width measurements. These Phase II data for the flow width measurements (in micrometres, *μ*m) of a hard-bake process in a semiconductor manufacturing are given in Table 4. This table displays the sample means, ${\bar{X}}_{i}$, for the 15 samples of flow width measurements. The estimates of the mean and standard deviation of the flow width measurements are established from the Phase I data to be ${\hat{\mu }}_{0}$ = 1.5 and $\hat{\sigma }$ = 0.15, respectively.

**Table 4:** Flow width measurements (*μ*m) for the hard-bake process.

Sample number, *i*	Wafers					${\bar{X}}_{i}$	Cumulative time	*CRL*
	1	2	3	4	5			
1	1.4483	1.5458	1.4538	1.4303	1.6206	1.4998	1.0	
2	1.5435	1.6899	1.5830	1.3358	1.4187	1.5142	2.5	
3	1.5175	1.3446	1.4723	1.6657	1.6661	1.5332	4.0	
4	1.5454	1.0931	1.4072	1.5039	1.5264	1.4152	5.5	
5	1.4418	1.5059	1.5124	1.4620	1.6263	1.5097	6.0	
6	1.4301	1.2725	1.5945	1.5397	1.5252	1.4724	7.5	
7	1.4981	1.4506	1.6174	1.5837	1.4962	1.5292	9.0	
8	1.3009	1.5060	1.6231	1.5831	1.6454	1.5317	10.5	
9	1.4132	1.4603	1.5808	1.7111	1.7313	1.5793	12.0	
10	1.3817	1.3135	1.4953	1.4894	1.4596	1.4279	12.5	
11	1.5765	1.7014	1.4026	1.2773	1.4541	1.4824	13.0	
12	1.4936	1.4373	1.5139	1.4808	1.5293	1.4910	14.5	
13	1.5729	1.6738	1.5048	1.5651	1.7473	1.6128	16.0	
14	1.8089	1.5513	1.8250	1.4389	1.6558	1.6560	16.5	14
15	1.6236	1.5393	1.6738	1.8698	1.5036	1.6420	17.0	1

It is assumed that the desired *ATS*_0_ and δ_opt_ values are 200 and 1, respectively. The limits of the $\bar{X}$ sub-chart are computed based on Eq (20A), (20B), (20C) and (20D) using the estimates ${\hat{\mu }}_{0}$ = 1.5 and $\hat{\sigma }$ = 0.15 from the Phase I data. The length of the initial sampling interval *t*_*f*_ = 1 hour is considered. The length of the short sampling interval decided by the $\bar{X}$ and *CRL* sub-charts is fixed as 0.5 hour (*d*_1_ = *d*_3_ = 0.5). The length of the long sampling interval decided by the $\bar{X}$ sub-chart is fixed as 1.5 hours (*d*_2_ = 1.5). Then the length of the long sampling interval decided by the *CRL* sub-chart is calculated by letting *E*(*T*_*CRL*_) = 1. The optimal parameters *L*_1_, *L*_2_, *k*, *w* and *d*_4_ for the VSI synthetic $\bar{X}$ chart are found to be 43, 3, 2.04, 0.64 and 3.25, respectively, using the procedure enumerated in Section 4.

**Fig 4:**
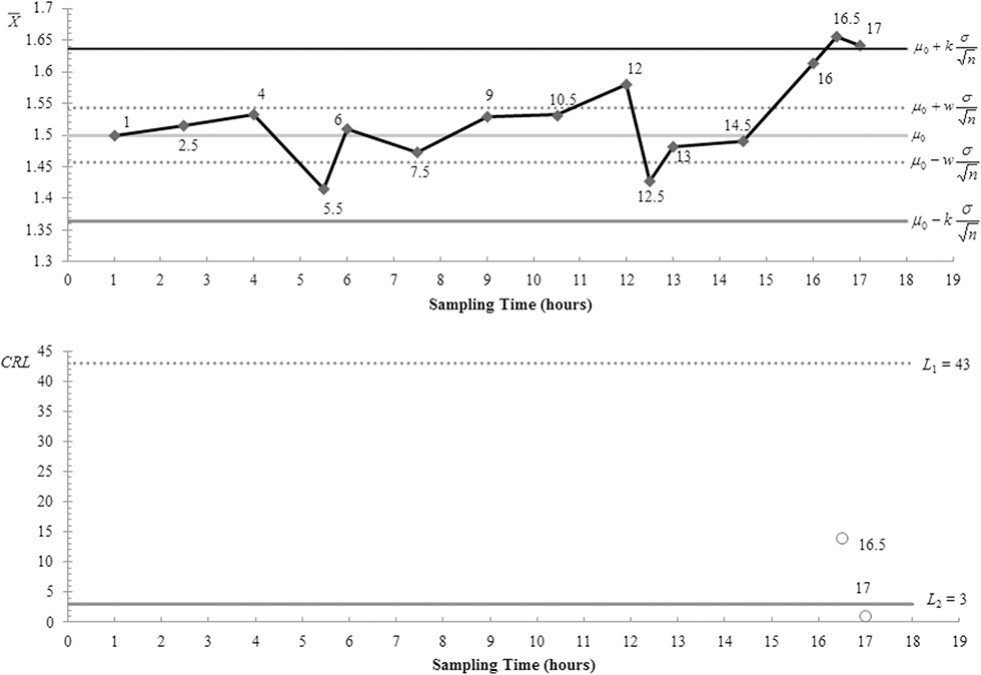
The VSI synthetic $\bar{X}$ chart for the flow width measurements. This chart is used to monitor the flow width measurements (in micrometres, *μ*m) for the hard-bake process. The chart signals an out-of-control at the 15^th^ sample which corresponds to the 17^th^ hour from the start of the process.

Fig 4 plots the ${\bar{X}}_{i}$ samples in Table 4 on the VSI synthetic $\bar{X}$ chart. The values beside the sample points on the VSI $\bar{X}$ and *CRL* sub-charts are the times when samples are taken. For the sake of explanation, consider *i* = 1. Here, the 1^st^ sample is taken after *t*_*f*_ = 1 hour. As sample 1 falls in the control region of the VSI $\bar{X}$ sub-chart, sample 2 is taken after the long sampling interval *d*_2_ = 1.5 hours. Then as sample 2 also falls in the control region of the VSI $\bar{X}$ sub-chart, sample 3 is obtained after *d*_2_ = 1.5 hours. The process of deciding the sampling interval length continues until sample 4 which falls in the warning region. Therefore, sample 5 is taken after the short sampling interval *d*_1_ = 0.5 hour. This process continues until the VSI synthetic $\bar{X}$ chart signals an out-of-control at the 15^th^ sample (as the *CRL* associated with *i* = 15 is less than *L*_2_) which corresponds to 17 hours from the start of process monitoring.

## Conclusions

The VSI synthetic **$\bar{X}$** chart is proposed in this paper. An optimal design procedure is presented by minimizing the out-of-control *ATS* for a desired size of mean shift, where a quick detection is required, based on the specified sample size, *n* and *ATS*_0_. This optimization procedure simplifies the design of the VSI synthetic $\bar{X}$ chart and facilitates its use among practitioners and engineers in manufacturing. Table 1 provides some optimal charting parameters for selected (*ATS*_0_,δ_opt_,*n*,*d*_1_,*d*_2_,*d*_3_) combinations for the VSI synthetic $\bar{X}$ chart, in order to facilitate a quick implementation of the proposed chart in manufacturing. The optimization program can be requested from the first author to enable a quick computation of the optimal charting parameters if other (*ATS*_0_,δ_opt_,*n*,*d*_1_,*d*_2_,*d*_3_) combinations are desired. An illustrative example is also provided to explain the chart’s construction to practitioners.

The VSI type charts have found applications in various fields. For example, the VSI *np* charts was used to improve the effectiveness of detecting small or moderate process shifts in the ceramic substrate production line [33]. The VSI $\bar{X}$ chart was also applied in the textile manufacturing company to monitor the tensile strength of a fibre used in producing cloth [34]. The VSI EWMA was employed to monitor linear calibration profiles for optical imaging system [35]. Ou et al. [36] explained the application of the VSI sequential probability ratio test (SPRT) chart in three different case studies. In the first case study, the VSI SPRT chart was employed to monitor the thickness of the silicon dioxide layer for a semiconductor component in a semiconductor company. In the second case study, the VSI SPRT chart was used to monitor the breaking strength of a nylon fibre while in the third study, the VSI SPRT chart was applied to monitor the diameter of a special drill produced by a tool work factory. Like other VSI type charts, the VSI synthetic $\bar{X}$ chart is also applicable in the above applications.

It is found that generally the VSI synthetic $\bar{X}$ chart performs better than the $\bar{X}$, synthetic $\bar{X}$ and VSI $\bar{X}$ charts for detecting all sizes of shifts. However, the EWMA $\bar{X}$ chart outperforms the VSI synthetic $\bar{X}$ chart for detecting small shifts but the latter prevails for detecting moderate and large shifts. Lastly, further research can be made to investigate the construction of other types of adaptive synthetic $\bar{X}$ charts, such as the VSS synthetic $\bar{X}$, VSSI synthetic $\bar{X}$ and VP synthetic $\bar{X}$ charts and their multivariate counterparts.
